# Sternal Foramina Detected by Postmortem Computed Tomography in the Japanese Population: Prevalence and Developmental Patterns

**DOI:** 10.1155/rrp/4298982

**Published:** 2025-09-30

**Authors:** Akihito Usui, Sonoka Sato, Eiji Suzuki, Sohtaro Mimasaka, Tomohiro Kaneta

**Affiliations:** ^1^Department of Diagnostic Image Analysis, Tohoku University Graduate School of Medicine, Sendai, Miyagi, Japan; ^2^Department of Forensic Medicine, Tohoku University Graduate School of Medicine, Sendai, Miyagi, Japan

**Keywords:** foramina, forensic radiology, postmortem computed tomography, sternal ossification, sternum

## Abstract

**Background:**

Sternal foramina are congenital anomalies arising from incomplete fusion of sternal ossification centers. They are often clinically silent but can pose risks during sternal procedures because of their proximity to critical mediastinal structures. Large-scale postmortem computed tomography (CT) studies of their prevalence in Japanese populations are limited, and their developmental origins remain elusive. We aimed to investigate the development, prevalence, and anatomical characteristics of sternal foramina in a large Japanese cohort using postmortem CT.

**Methods:**

We retrospectively reviewed postmortem CT scans from 1503 adults (1021 males, 482 females; age range: 20–96 years) and 92 pediatric cases (age range: 0–8 years). In adults, we assessed prevalence, sex distribution, location, diameter, and adjacent structures. In pediatrics, ossification patterns of the third and fourth sternebral segments were evaluated to explore developmental contributions to foramen formation.

**Results:**

Sternal foramina were present in 3.7% of adults. They were more frequent in males (4.3%) than in females (2.5%), although the difference was insignificant. Most foramina were located at the level of the fifth costal notch and overlaid the pericardium or lung in 72% of evaluable cases. The median diameter was 4.5 mm. In pediatric cases, 11 (12%) exhibited lower-sternebral ossification-center patterns that could form sternal foramina, supporting a developmental origin. An estimated 31% of these patterns may persist into adulthood with unfused segments.

**Conclusion:**

Sternal foramina occurred in 3.7% of adults and were often situated over vital structures, posing procedural risks. Among pediatrics, ossification patterns that may impede fusion—defined as horizontal two-center or ≥ 3 center configurations—were present in 12%, and approximately 31% of these patterns appear to persist into adulthood as sternal foramina. These findings support a developmental basis for sternal foramina and emphasize the importance of recognizing them during imaging and procedural planning.

## 1. Introduction

**Figure 1:**
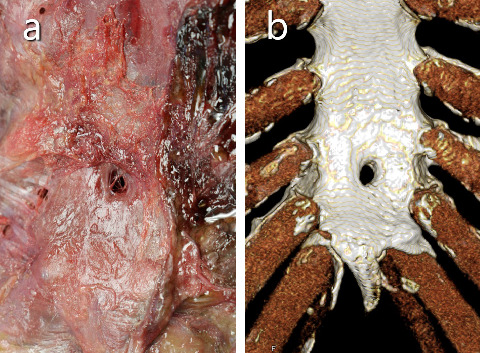
Dorsal view of the sternal foramen obtained at autopsy (a) and a volume-rendered dorsal view of the sternal foramen reformatted from computed tomography data of the same case (b).

Sternal foramina are congenital anatomical variants that arise because of incomplete fusion of ossification centers during sternal development [1]. They are frequently asymptomatic and are typically discovered incidentally through imaging or autopsy (Figure 1), but they have significant clinical relevance [2–4]. The presence of a sternal foramen can pose risks during invasive procedures such as acupuncture, sternal puncture, or bone marrow biopsy, as inadvertent injury to underlying vital structures may result in fatal complications [2, 5]. With the widespread adoption of multidetector computed tomography (MDCT), the detection and characterization of such variants have improved, enabling more detailed assessment of their location and morphology [3]. These foramina are often located in the lower portion of the sternal body and may overlie the pericardium or lungs [6]. Recent imaging studies have emphasized their morphometric parameters and variable prevalence across populations [7]. Furthermore, MDCT has proven valuable for adults and for evaluating sternal ossification patterns in children [8]. However, comprehensive postmortem CT-based analyses in Japanese populations remain limited [9], and the developmental mechanisms that contribute to persistent sternal foramina in adults are not fully understood. Early anatomical studies suggested that the pattern and timing of ossification may influence the final morphology of the sternum [10]. Meta-analytic data further reinforce that sternal foramina are often benign, but their clinical implications merit careful attention [11]. Their frequency and position are especially important in both diagnostic imaging and interventional planning [12, 13], and recent efforts have been targeted at establishing safer procedural zones based on CT imaging findings [14].

Despite the increasing reports on the prevalence and morphology of sternal foramina using MDCT in living populations, large-scale studies based on postmortem CT remain limited, especially for the Japanese population. Given that postmortem imaging provides a unique opportunity to evaluate anatomical variants without motion artifacts or radiation constraints, its underutilization represents a notable gap in the anatomical literature. Furthermore, prior developmental studies have proposed that segmentation anomalies in the lower sternebrae (the segmental bones that make up the body of the sternum in early development) may contribute to foramen formation [10], but only a few investigations have attempted to directly correlate pediatric ossification patterns with the presence of sternal foramina in adults.

The present study aimed to investigate the prevalence and anatomical characteristics of sternal foramina in a large Japanese cohort using postmortem CT. It also aimed to explore their developmental origins by analyzing ossification patterns in pediatric cases with a focus on the number and distribution of ossification centers in the lower sternebrae. This dual approach was used to enhance the understanding of the clinical relevance and embryological basis of sternal foramina and improve diagnostic interpretation and procedural planning in clinical and forensic settings.

## 2. Materials and Methods

### 2.1. Study Design and Ethical Approval

This retrospective, cross-sectional study analyzed postmortem CT data to assess the prevalence and anatomical characteristics of sternal foramina in adults and evaluate sternal ossification patterns in pediatric cases. All postmortem CT scans were obtained at the Autopsy Imaging Center of our institution between 2009 and 2024. The study was approved by the appropriate institutional ethics committee (Approval No. 2024-1-712). As the study involved deceased individuals, informed consent was not applicable. However, information about the study was disclosed to next of kin through an opt-out procedure in accordance with relevant ethical guidelines.

### 2.2. Study Population

#### 2.2.1. Adults

The adult dataset included 1503 postmortem cases (1021 males and 482 females; age range: 20–96 years) collected between 2017 and 2024. The inclusion criterion was complete visualization of the sternum on CT. Of 1781 patients aged 20 years or older over 7 years, 278 with extensive trauma, severe sternal deformity, advanced decay with skeletal defects, or imaging artifacts that interfered with morphological evaluation were excluded.

#### 2.2.2. Children

The pediatric dataset comprised 92 postmortem cases (50 boys, 42 girls; age range: 0–8 years) collected between 2009 and 2024. Only cases with unfused sternebrae were included. Among these, 21 cases with sufficient preservation of the fourth sternebral segment were eligible for analysis of ossification patterns.

#### 2.2.3. CT Protocol and Image Reconstruction

All scans were performed using a 64-slice multidetector CT scanner (Aquilion 64; Canon Medical Systems Corp., Otawara, Japan). The tube voltage was set at 120 or 135 kV for adults and 120 kV for pediatric cases. Automatic exposure control was applied to all scans. Slice thickness ranged from 0.5 to 2.0 mm for adult cases and 0.5–1.0 mm for pediatric cases.

Bone reconstruction filters were not applied to chest CT images of all pediatric cases because bone evaluation was not originally the main objective. Therefore, images of six cases were evaluated using soft tissue filters. Multiplanar reconstruction (MPR), maximum intensity projection (MIP), and volume rendering were employed for morphological evaluation. All reconstructions were performed using a medical image visualization and analysis workstation (Ziostation2, version 2.4.3.3; Ziosoft Inc., Tokyo, Japan), and image quality was verified for diagnostic adequacy.

### 2.3. Image Analysis

#### 2.3.1. Adult Sternal Foramina

**Figure 2:**
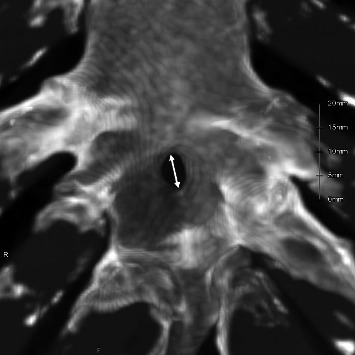
Maximum diameter of the hole measured from the maximum intensity projection image (double arrow indicates the maximum diameter).

For adults with sternal foramina, the following parameters were evaluated: prevalence and sex distribution; anatomical location in the body of the sternum (based on costal cartilage or intercostal level where the center of the foramen is located); maximum diameter (measured with MIP image; the image at the time of measurement is shown in Figure 2); and correlation between diameter and age and comparison across anatomical locations.

**Figure 3:**
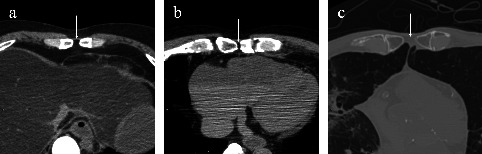
(a) Computed tomography image of a case of adipose tissue present immediately below the sternal foramen (indicated by the arrow). The pericardium can be seen in (b), and the lungs in (c). These are postmortem images of different bodies.

Axial images were used to identify underlying structures directly beneath each foramen (such as pericardium, lung, liver, or fat) (Figure 3). Thirteen cases were excluded from this subanalysis because of decomposition or severe skeletal deformity. Additional anatomical variants were recorded. These included foramina and bifid morphology of the xiphoid process; foramina of the manubrium; concurrence of multiple anatomical variants; and bifid ribs assessed on axial and coronal images. All adult CT datasets were reviewed independently by two fourth-year radiography students using prespecified operational definitions (presence of a foramen, level assignment by costal landmark, and maximal diameter). Discrepancies between the two initial readers were recorded and resolved by consensus with senior radiographers with more than 20 years of clinical experience. The consensus read served as the final dataset for analysis.

#### 2.3.2. Pediatric Ossification Patterns

For the 21 pediatric cases suitable for analysis, the number and distribution of ossification centers within the third and fourth sternebral segments were recorded. Separate ossification centers were defined as distinct foci of calcification separated by noncalcified cartilage on axial CT (window width: 3000 HU; window level: 250 HU). Evaluations were performed on coronal MPR images. All cases were independently reviewed by two fourth-year radiography students. Any discrepancies were resolved by consensus with experienced radiographers with over 20 years of clinical practice.

#### 2.3.3. Statistical Analysis

All statistical analyses were conducted using JMP Pro, version 17.2 (SAS Institute Inc., Cary, NC, USA). Sex-related differences in prevalence were assessed using the chi-squared test. Differences in foramen diameter related to sex were analyzed using the Wilcoxon rank-sum test. Correlations between age and foramen diameter were evaluated using Pearson's correlation coefficient. Differences in foramen diameter related to anatomical location were compared using the Steel–Dwass test for multiple comparisons.

*p* values of < 0.05 denoted statistical significance. In addition to *p* values, we report point estimates with 95% confidence intervals (95% CIs) to enhance interpretability. For proportions, binomial 95% CIs were computed using the Wilson score method; for correlations, Pearson's correlation coefficient (*r*) was reported with 95% CIs based on Fisher's *z* transformation. For medians, interquartile ranges (IQRs) were reported. For between-sex comparisons of prevalence, we report the risk ratio with 95% CIs calculated on the log scale (back-transformed), alongside the risk difference with Newcombe CIs.

## 3. Results

A total of 1503 adult and 92 pediatric postmortem CT cases were analyzed. Of the pediatric cases, 21 were eligible for the evaluation of lower-sternebral ossification patterns.

### 3.1. Prevalence and Morphological Characteristics of Sternal Foramina in Adults

Sternal foramina were identified in 56 of 1503 adults, corresponding to a prevalence of 3.7% (95% CI 3.0%–4.7%). The prevalence was higher for males (44/1021; 4.3% [95% CI 3.2%–5.7%]) than for females (12/482; 2.5% [95% CI 1.4%–4.3%]), although the difference was not statistically significant (*p*=0.1479, chi-squared test). The prevalence risk difference (male–female) was 1.8% points (95% CI −1.1 to 4.3), and the risk ratio was 1.73 (95% CI 0.92–3.25); neither estimate was statistically significant.

Of the 56 foramina, 5 (9%) were located cranial to the fourth intercostal space, 37 (66%) were at the fifth costal notch, 12 (21%) were at the fifth intercostal space, and 2 (4%) were at or below the sixth costal notch. With the exception of one foramen at the fourth costal notch, all were situated in the lower half of the sternal body.

**Figure 4:**
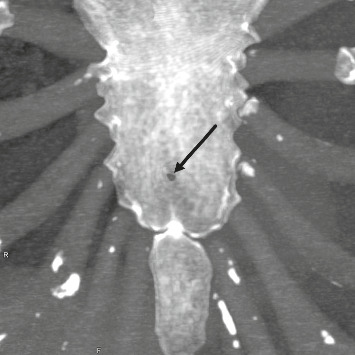
Maximum intensity projection image of a 38-year-old woman with a sternal foramen (indicated by arrow) at the level of the fifth costal notch with a minimum diameter of 1.5 mm.

**Figure 5:**
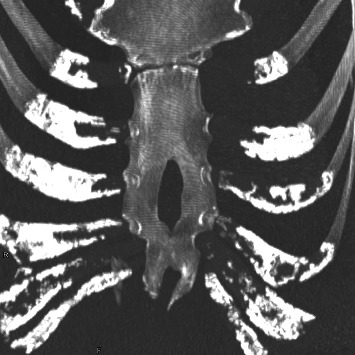
Maximum intensity projection image of a 52-year-old woman who has a sternal foramen at the fourth intercostal level with a maximum diameter of 26 mm and a bifid xiphoid.

**Figure 6:**
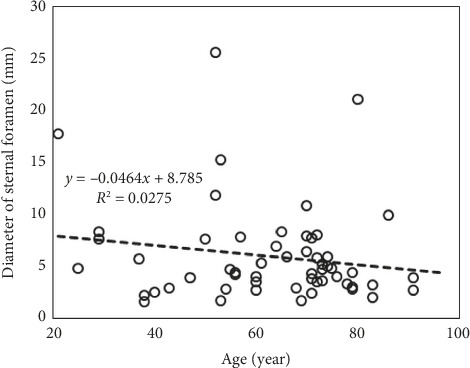
Relationship between age and sternal foramen diameter.

The maximum diameter ranged from 1.5 to 26 mm (Figures 4 and 5) with a median value of 4.5 mm (IQR 3.2–7.5). The median diameter was slightly larger in males (4.7 mm [IQR 3.4–7.5]) than in females (3.9 mm [IQR 2.7–4.9]), but this difference was not statistically significant (*p*=0.8385, Wilcoxon rank-sum test). A weak negative correlation between diameter and age was observed (*r* = −0.17 [95% CI −0.41–0.10] Pearson's correlation; *R*^2^ = 0.028 coefficient of determination), indicating no meaningful age-dependent trend (Figure 6).

In addition, the foramina were grouped into three according to their anatomic location: above the fourth intercostal space (*n* = 5), at the fifth costal notch (*n* = 37), and at or below the fifth intercostal space (*n* = 14). The median maximum diameters of the sternal foramen were 2.9, 4.3, and 4.8 mm for the three groups, respectively. No significant differences were found in the maximum diameter of the sternal foramen between these groups (Steel–Dwass test, *p* > 0.38 for all comparisons).

### 3.2. Underlying Structures and Associated Anatomical Variants

Of the 56 adults with sternal foramina, 43 had sufficient preservation to allow assessment of the structures directly underlying the foramen. The most commonly observed adjacent structures were the pericardium, lung, adipose tissue, and liver in 16 (37%; 95% CI 24%–52%), 15 (35%; 95% CI 22%–50%), 9 (21%; 95% CI 11%–35%), and 3 (7%; 95% CI 2.4%–19%) cases, respectively.

Additional sternal variants were recorded for the overall adult population (*n* = 1503). Foramina were present in the xiphoid process in 363 cases (24%; 95% CI 22%–26%). A bifid xiphoid morphology was observed in 416 cases (28%; 95% CI 25%–30%). A foramen in the manubrium was observed in one case (0.067%; 95% CI 0.010%–0.38%). Fourteen individuals (0.93%; 95% CI 0.56%–1.6%) had both sternal body and xiphoid foramina. One case had a concurrent sternal foramen and a bifid rib (0.067%; 95% CI 0.01%–0.38%).

### 3.3. Ossification Patterns in Pediatric Cases

**Figure 7:**
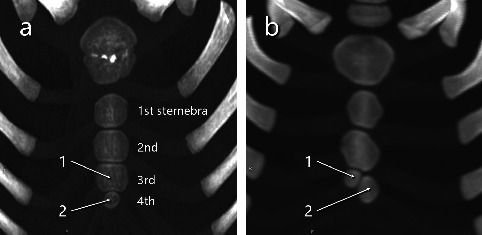
Two maximum intensity projections with two ossification centers (indicated by arrows and numbers) in the third and fourth sternebrae. (a) A 6-year-old girl. (b) An 11-month-old boy.

**Figure 8:**
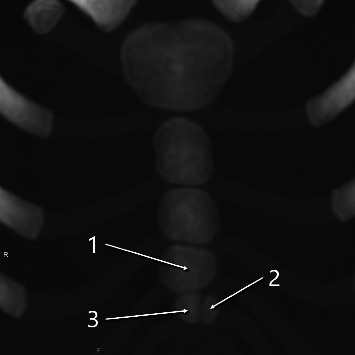
Maximum intensity projection image of a 3-year-old girl with three ossification centers (indicated by arrows and numbers) in the third and fourth sternebrae.

**Figure 9:**
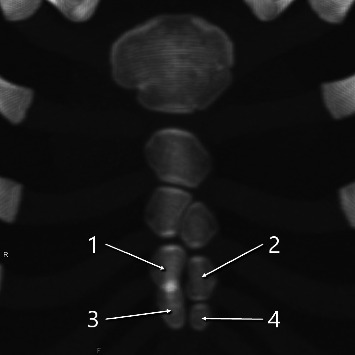
Maximum intensity projection image of a 3-year-old boy with four ossification centers (indicated by arrows and numbers) in the third and fourth sternebrae.

Of the 92 pediatric cases (ages 0–8), the fourth sternebral segment was preserved in 21 for ossification pattern analysis. Of these 21 cases, 13 had two ossification centers across the third and fourth segments—10 with vertical (superior–inferior) alignment (Figure 7(a)) and 3 with horizontal (left–right) alignment (Figure 7(b)); 7 had three centers (Figure 8); and 1 had four centers (Figure 9) (Table 1). Of the 21 pediatric cases, 11 (52.4%) had multiple ossification centers—specifically, two centers with horizontal alignment (*n* = 3), three centers (*n* = 7), or four centers (*n* = 1)—that may impede complete midline fusion and potentially contribute to sternal foramen formation in later life. The remaining 10 cases (47.6%) had two centers with vertical alignment, which is less likely to result in sternal foramina. This corresponds to 11/92 (12.0%) of all pediatric cases.

## 4. Discussion

Using a large postmortem CT cohort, we contextualized prevalence, anatomic distribution, and developmental patterns of sternal foramina in relation to prior CT-based literature, procedural risk mapping, and pediatric ossification data. Below, we discuss these findings in light of previous reports and their clinical and forensic implications.

The adult prevalence observed in this study is consistent with previous CT-based studies reporting rates between 3.1% and 13.0% across different populations [1–3, 11]. A recent meta-analysis revealed population-level variation, with incidence rates of 6.2% in North America, 8.6% in Europe, 13.9% in South America, and 13.6% in Africa [11]. The 3.7% prevalence in this Japanese cohort falls within the lower end of this spectrum.

As described in earlier studies, sternal foramina are typically asymptomatic but clinically relevant because of the potential for them to be mistaken for lytic lesions or traumatic injuries on imaging [2, 4, 6] and because of their associated risk during sternal procedures [5, 12, 14]. In this study, the majority of foramina were located at the fifth costal notch, which is consistent with earlier reports emphasizing the lower sternal body as a common site [2, 3, 9].

The median maximum transverse diameter of the foramina was 4.5 mm, with no significant differences related to sex or age. This contrasts with several prior studies reporting larger diameters for males [2, 9, 12], possibly because of the population (forensic autopsy cases only or Asian race only) or methodological differences. Additionally, no significant correlation was found between diameter and anatomical location, although more inferior sternal foramina tended to be larger. This supports the notion that location is more developmentally determined than morphometrically patterned, although the details are unclear. A comparative summary of CT-based prevalence and morphometry across populations is provided in Table 2, showing that our prevalence lies at the lower end of reported ranges, with similar caudal predilection and mixed evidence on sex-related diameter differences.

The finding that 37% of foramina were positioned directly over the pericardium and 35% over the lung underscores the potential procedural hazards associated with sternal puncture, acupuncture, or intraosseous access [5, 12, 14, 15]. Previous studies have emphasized the risk of fatal cardiac tamponade following sternal penetration through a congenital foramen [5]. Ma et al. [7] demonstrated a positive correlation between subcutaneous fat thickness and skin-to-pericardium distance, suggesting the possibility of using soft tissue measurements as risk predictors for procedural injury [13]. Patterns of directly adjacent structures in our postmortem cohort broadly align with prior CT series but with a relatively higher share of pericardial contact. In a living-cohort study, lung adjacency predominated (53%), followed by mediastinal fat (33%) and pericardium/heart (20%) [15]. A larger living-cohort analysis reported mediastinal adipose tissue in 37%, lung in 34%, pericardium in 16%, and “both” tissues in 13% [7]. By comparison, we observed pericardium in 37% (16/43), lung in 35% (15/43), fat in 21% (9/43), and liver in 7% (3/43). This study was a postmortem series. We did not control lung inflation or postmortem interval, and postmortem changes (e.g., atelectasis and fluid shifts) may influence the classification of adjacent structures, limiting direct comparability with living-cohort CT studies. A thorough knowledge of human morphology is essential for the proper interpretation and diagnosis of radiological images, as well as for planning medical interventions and forensic identification [16].

Based on the anatomical distribution observed in this study, sternal interventions should be avoided at or below the fifth costal level when a foramen cannot be excluded. This is consistent with previous recommendations that identify the fourth through sixth costal levels as a high-risk region for blind sternal procedures [14].

**Figure 10:**
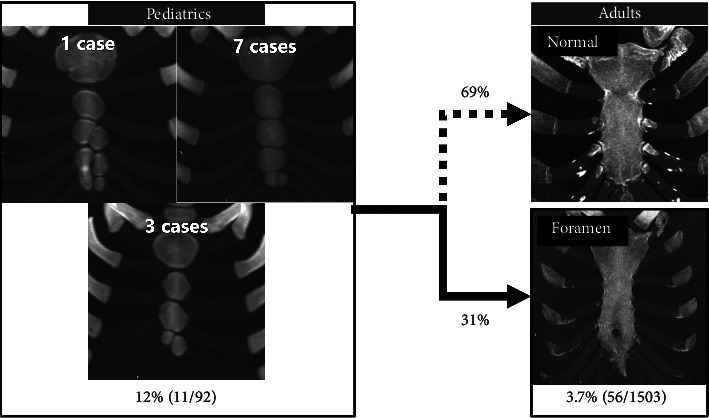
Conceptual diagram showing the estimated proportion of pediatric ossification patterns—defined as two-center or ≥ 3-center configurations—that persist as adult sternal foramina. In our cohort, approximately 31% persist, whereas about 69% likely achieve complete midline fusion.

**Figure 11:**
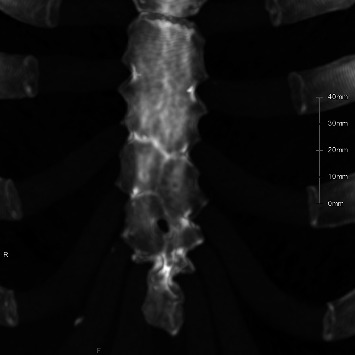
Maximum intensity projection image of a 25-year-old woman with a few residual fused lines located in the third and fourth segments in the lower sternum.

Of 21 evaluable pediatric cases, 11 (52.4%) had a pattern of ossification centers that could form a sternal foramen. This corresponds to 11/92 (12.0%) of all pediatric cases. These findings support the developmental hypothesis that increased segmentation in the lower mesosternum may interfere with midline fusion, contributing to the persistence of sternal foramina into adulthood [10, 13]. The ossifications capable of forming these fourth and fifth intercostal foramen variants were found in approximately 12% of pediatric cases. Only an estimated 31% of these are thought to result in persistent sternal foramina, while the remaining 69% likely lead to complete midline fusion (Figure 10). In this study, we would expect 180 foramina in our adult cohort based on the assumption that 11 out of 92 children with two or more multiple ossification centers in the inferior sternum (Figures 7(b), 8, 9) all develop sternal foramina at the 4th and 5th intercostal levels when they grow up. However, we only observed 56 foramina. This means that only approximately 31% of the expected foramina manifested in adulthood within our observed cohort. This suggests that the presence of multiple ossification centers may be a necessary but not sufficient condition, and other factors—such as fusion timing, local biomechanical forces, or individual variability—may also influence foramen persistence. In adults, cranial or 5^th^-level locations were uncommon—5/56 (9%) above the 4^th^ intercostal space and 12/56 (21%) at the 5^th^ intercostal space—mirroring the low frequency of pediatric horizontal two-center patterns (3/21, 14.3%). The horizontal two-center configuration plausibly reflects a midline defect within a single sternebral level and may therefore underlie the less frequent foramina at the 5^th^ costal level (notch or interspace) or at more cranial sites observed in adults. Given the small numbers on both sides (horizontal two-center in children; cranial/5^th^-level foramina in adults), this developmental link should be regarded as hypothesis-generating rather than definitive. As shown in Figure 11, we experienced only one case in an adult (25-year-old female) in which traces of fusion remained in the sternal body and sternal foramen formation was evident. This is only one case, and it does not constitute strong evidence. However, it may indicate that fusion of ossification centers is related to sternal foramen formation.

Additionally, this study documented several other anatomical variants, including foramina and bifurcations of the xiphoid process (24%–28%), a rare case of a manubrial foramen (0.067%), and a bifid rib. These findings are of forensic and radiological relevance, as they may mimic pathological lesions or traumatic injuries [4, 6, 11]. Rare anatomical variants, if recorded antemortem, may also serve as valuable indicators for personal identification [17, 18].

Taken together, these findings provide empirical support for the hypotheses proposed in the Introduction. By integrating adult morphological data with developmental patterns in pediatric cases, this study provides new insights into the etiology of sternal foramina and underscores their relevance in both clinical practice and forensic investigations.

This study has several limitations. First, the analysis was retrospective and based solely on forensic autopsy cases. Therefore, the sample may not be fully representative of the general living population, especially in terms of age distribution, underlying health status, and causes of death. These may introduce selection bias and potentially influence the observed prevalence, morphology, and developmental implications of sternal foramina. Further, postmortem changes such as decomposition may have affected image interpretation for a few cases, despite our exclusion of severely degraded scans.

## 5. Conclusion

The three key findings were as follows:

• Prevalence: Sternal foramina were present in 3.7% of adults (95% CI 3.0%–4.7%), with a nonsignificant male–female difference (4.3% vs 2.5%).
• Anatomy and risk: Foramina predominantly involved the lower sternal body—mostly the 5th costal notch—with a median diameter of 4.5 mm and frequent overlap with the pericardium or lung (72%), underscoring procedural risk.
• Developmental origin: Multiple ossification centers in the lower sternebrae were observed in ∼12% of pediatric cases, and only a subset (∼31%) appear to persist into adulthood, supporting a developmental basis for sternal foramina.

These findings emphasize the need for awareness and preprocedural imaging review to minimize complications and improve diagnostic interpretation.

## Figures and Tables

**Table 1:** Distribution and alignment of ossification centers in the third and fourth lower sternebral segments among 21 pediatric postmortem computed tomography cases.

Ossification pattern (in lower sternebrae)	No. of cases	Percentage (%)
Patterns that may lead to foramen formation	11	52.4
2 centers—horizontal alignment (left–right)	3	14.3
3 centers	7	33.3
4 centers	1	4.8
Patterns less likely to form foramina	10	47.6
2 centers—vertical alignment (superior–inferior)	10	47.6
Total	21	100

**Table 2:** Comparative CT studies of sternal foramina across populations.

Study	Population (*n*)	Prevalence (%)	Predominant location	Diameter, mm (central tendency)^∗^	Sex-related difference in diameter	Study type/modality
Present study (Japan)	1503	3.7	Lower half of sternal body, mostly 5^th^ costal notch	4.5 [median]	No significant difference (*p*=0.8385)	Postmortem CT
Yekeler et al. [1] (Turkey)	1000	4.5	Lower sternal body	6.5 [mean]	Not reported	MDCT
Babinski et al. [12] (Brazil)	114	10.5	Inferior part of sternal body	5.5 [mean]	Not reported	MDCT
Vulovic et al. [16] (Serbia)	422	5.9	Distal half of sternal body	3.9 × 4.2 [mean]	No significant difference (*p* > 0.05)	MDCT
Ma et al. [7] (China)	2500	4.4	Mostly the 5^th^ costal cartilage space	6.0 × 6.8 [mean]	Larger in males	MSCT
Pasieka et al. [11] (meta-analysis)	16,666	13.6 (Africa), 7.5 (Asia), 8.6 (Europe), 6.2 (North America), 13.9 (South America)	Mostly the 5^th^ intercostal segment	4.7 × 5.6 [mean]	Not reported	Cadaveric + MDCT/CT/X-ray

*Note:* MSCT, multislice CT.

^∗^Central tendency reported by each study: present study = median maximum diameter; others report means (single value or horizontal × vertical).

Abbreviations*:* MDCT, multidetector computed tomography.

## Data Availability

The data that support the findings of this study are available from the corresponding author upon reasonable request.

## References

[1] Yekeler E., Tunaci M., Tunaci A., Dursun M., Acunas G. (2006). Frequency of Sternal Variations and Anomalies Evaluated by MDCT. *American Journal of Roentgenology*.

[2] Choi P. J., Iwanaga J., Tubbs R. S. (2017). A Comprehensive Review of the Sternal Foramina and Its Clinical Significance. *Cureus*.

[3] Ishii S., Shishido F., Miyajima M. (2011). Causes of Photopenic Defects in the Lower Sternum on Bone Scintigraphy and Correlation With Multidetector CT. *Clinical Nuclear Medicine*.

[4] Restrepo C. S., Martinez S., Lemos D. F. (2009). Imaging Appearances of the Sternum and Sternoclavicular Joints. *RadioGraphics*.

[5] Halvorsen T. B., Anda S. S., Naess A. B., Levang O. W. (1995). Fatal Cardiac Tamponade After Acupuncture Through Congenital Sternal Foramen. *The Lancet*.

[6] McCormick W. F. (1981). Sternal Foramena in Man. *The American Journal of Forensic Medicine and Pathology*.

[7] Ma D., Wang J., Wang Z.-H., Cui X. (2024). Sternal Foramina: An Imaging Study. *Clinical Anatomy*.

[8] Gumeler E., Akpinar E., Ariyurek O. M. (2019). MDCT Evaluation of Sternal Development. *Surgical and Radiologic Anatomy*.

[9] Vatzia K., Fanariotis M., Makridis K. G., Vlychou M., Fezoulidis I. V., Vassiou K. (2021). Frequency of Sternal Variations and Anomalies in Living Individuals Evaluated by MDCT. *European Journal of Radiology*.

[10] Ashley G. T. (1956). The Relationship Between the Pattern of Ossification and the Definitive Shape of the Mesosternum in Man. *Journal of Anatomy*.

[11] Pasieka P., Pasieka P. M., Komosa A. (2023). Prevalence and Morphometry of Sternal and Xiphoid Foramen: A Meta-Analysis on 16,666 Subjects. *Surgical and Radiologic Anatomy*.

[12] Babinski M. A., de Lemos L., Babinski M. S. D., Gonçalves M. V. T., De Paula R. C., Fernandes R. M. P. (2015). Frequency of Sternal Foramen Evaluated by MDCT: A Minor Variation of Great Relevance. *Surgical and Radiologic Anatomy*.

[13] Turkay R., Inci E., Ors S., Nalbant M. O., Gurses I. A. (2017). Frequency of Sternal Variations in Living Individuals. *Surgical and Radiologic Anatomy*.

[14] Boruah D. K., Prakash A., Yadav R. R. (2016). The Safe Zone for Blinded Sternal Interventions Based on CT Evaluation of Midline Congenital Sternal Foramina. *Skeletal Radiology*.

[15] Gossner J. (2013). Relationship of Sternal Foramina to Vital Structures of the Chest: A Computed Tomographic Study. *Anatomy Research International*.

[16] Vulovic M., Zivanovic-Macuzic I., Jeremic D. (2019). Multidetector Computed Tomography (MDCT) Estimation of Prevalence and Anatomic Characteristics of the Sternal Body Foramen in the Population of Central Serbia. *Vojnosanitetski Pregled*.

[17] Singh J., Pathak R. K. (2013). Sex and Age Related Non-Metric Variation of the Human Sternum in a Northwest Indian Postmortem Sample: A Pilot Study. *Forensic Science International*.

[18] Paraskevas G. K., Tzika M., Natsis K. (2016). Double Sternal Foramina in a Dried Sternum: A Rare Normal Variant and Its Radiologic Assessment. *Surgical and Radiologic Anatomy*.

